# Philadelphia Academy of Surgery

**Published:** 1893-07

**Authors:** 


### SOCIETY REPORTS.

### PHILADELPHIA ACADEMY OF SURGERY.

MEETING APRIL 3, 1893.

The President, DR. WM. HUNT, in the chair.

DR. JOHN H. BRINTON read a paper entitled

### TWO CASES OF AMPUTATION AT SHOULDER JOINT IN WHICH WYETH'S PINS, TO CONTROL HEMORRHAGE, WERE USED.

CASE 1.—*Osteitis deformans of the leg, followed at the expiration of twenty-three years by sarcoma of the humerus; amputation at shoulder joint by the oval method; use of Wyeth's pins to control hemorrhage; death on the tenth day.*—E. B., aged forty-eight years; born in Massachusetts; publisher. About twenty-three years ago he noticed tenderness over the right tibia, increased by pressure, by severe or prolonged exercise, and by barometric changes. Various antirheumatic measures were employed, but without avail. The limb did not become much worse; he was able to go about and follow the business of his life for years. During this time he was not lame, but experienced a sense of weakness in the limb. To use his own expression, "he favored that leg." In the course of years the bones of the leg had gradually increased in thickness, and had become curved. About ten years ago he consulted the late Prof Agnew, who told him that he could do nothing to relieve his slight disability of the limb and that the affection was incurable. About three years since he consulted me, but I could add nothing to what had been already said, and could suggest no treatment.

In November, 1891, the patient consulted me for a fracture of the body of the left scapula. This resulted from a fall backward as he was descending from the step of a railroad car, scapula striking the edge of a projecting board or plank. This fracture healed rapidly and well.

In June, 1892, in jumping from a street car while in motion, and while his hand grasped the railing, he experienced great pain just below the right shoulder, and felt that his arm was broken. He came directly to my office. On examination I detected crepitus, diagnosticated fracture of the anatomical neck of the humerus, and treated him for that injury. Union took place quickly, and full use of the limb was obtained. The only noticeable feature in this injury was the occurrence of slight pain referred to the outside of the humerus, about the lower portion of the upper third. There was at that time no enlargement of the bone at this locality.

On September 19, 1892, the patient again consulted me, stating that a "lump" had appeared on the outside of the humerus at its upper part. I examined the arm and found distinct cylindrical enlargement of the humerus, obviously a sarcoma of the bone, and I stated this to the patient, advising him to consent to the removal of the limb at the shoulder joint, if the diagnosis should be confirmed by a preliminary incision. At the patient's request, Drs. Packard and John Ashhurst saw the case in consultation, and they agreed with me in the propriety of immediate operation. From a careful examination of the patient's entire clinical history, there was no doubt in our mind that the case was one of osteitis deformans, first described by Paget, and which had been followed, as is so often the case, by the development of a malignant growth.

The operation was fixed for October 5, 1892, but at the preliminary shaving of the axilla and preparation of the limb, or by the patient's lifting of the limb, fracture, which may fairly be regarded as spontaneous and non-traumatic, occurred, as was made evident at the time of operation, done in the presence and with the assistance of Drs. Keen, Ashhurst, Packard and others.

To prevent hemorrhage, the long steel pins of Prof. Wyeth were inserted by Dr. Keen, the anterior one transfixing the anterior axillary fold in front of the vessels, penetrating the tendon of the pectoralis major muscle, and emerging near the end of the acromion.

The posterior needle pierced the deltoid and emerged just below the acromion. By carrying the needle, especially the anterior one, well upward, the constricting rubber band was placed so high as not to prevent the rotation of the humeral head, or to interfere materially with its disarticulation.

This patient suffered very slight loss of blood at the time of the operation, and received but little shock. He reacted promptly and perfectly, and for several days did well, the wound uniting throughout. On the night between the fifth and sixth day the temperature rose to 104.5°, and a copious eruption, similar to that of measles, appeared on the abdomen and chest, and eventually invaded the extremities, and indeed the whole body. There was marked coryza, and the tongue became brown and dry. This condition resisted all treatment and the free use of antipyretics. As the eruption spread the temperature still rose, reaching 107.5° and 108°, and the patient died on the afternoon of the 15th of October, the tenth day after the operation. The intellect remained clear until within an hour or so of the end.

I cannot but regard the death as due to some form of septic infection not easy to determine.

It is unnecessary to add that antisepsis was observed in the treatment before, during and after the operation.

The specimens, showing the sarcoma of the shaft of the humerus, and the peculiar indented fracture of its head and anatomical neck, are before this Academy. I particularly desire the observation of the Fellows to the fracture, which appears to me to have resulted from violent impact of an infiltrated diseased caput humeri against the edge of the glenoid cavity.

CASE 2.—*Amputation at shoulder joint for enchondroma of humerus.*—The other case of shoulder amputation, in which I used Wyeth's pins was that of a boy (I. B.) from Vermont, ten years of age. Nearly a year previously a tumor, apparently an enchondroma, began to develop on the inner side of the humerus, close to the head of the bone. It eventually grew until it attained a diameter of two and a half inches. It was painless, but interfered with the joint motion by its bulk. The boy was brought to the clinic of the Jefferson hospital, and after consultation with my colleagues, I determined to remove the arm at the shoulder.

This was accordingly done on the 28th of November, Wyeth's pins being first introduced by my colleague, Professor Keen. The anterior pin was made to emerge three-quarters of an inch above the tip of the acromion. As a result the circular turns of the tubing rested on a somewhat higher level than in the preceding case. Perfect freedom of the joint was preserved, and its disarticulation was not impeded. Previous section of the bone with the saw, as diiected by Prof. Wyeth, was not necessary. A roller bandage was applied as a compress under the tubing and directly over the artery. Hemorrhage was thus perfectly prevented, and the removal of the limb, as in the former case, was practically a bloodless procedure. This boy recovered without accident.

I may state that in both these instances an Esmarch elastic bandage was applied previous to the insertion of the pins.

DR. W. W. KEEN reported

AN OVARIAN TUMOR WEIGHING ONE HUNDRED AND ELEVEN POUNDS REMOVED FROM A CHILD OF FIFTEEN, WHOSE WEIGHT WAS SIXTY-EIGHT POUNDS.

Miss B., of Benezett, Pa., was first seen by me at Driftwood, Pa., February 26, 1892, at the request of Dr. V. K. Corbett, of Caledonia. She was then fourteen years of age and had never menstruated. About eighteen months before I saw her her abdomen began to enlarge. Six months afterward Dr. Corbett was consulted for an attack of considerable pain in the left side of the abdomen. He found that she was only voiding eight ounces of urine in twenty-four hours, but under proper treatment this soon reached a quart in amount, and has remained so ever since. He never discovered any albumin in the urine. In October, 1891, she had been tapped by a gynecologist, who is said to have diagnosticated a solid and probably a malignant tumor, connected most likely with the liver, omentum and ovary, and who deemed its removal not feasible.

I found the abdomen enormously distended with fluid, and advised very strongly that a small incision should be made in the abdominal wall, so that I could determine the relations of the growth with accuracy. Her father, however, was not present, and had made it a condition that nothing beyond tapping should be done. I tapped her immediately and removed considerably over three gallons of amber-colored fluid. When this was evacuated I discovered a lobulated tumor on the right side of the abdomen, under the liver, and apparently attached to it. It was evidently cystic in part, there being at least two cysts perceptible. Each of these I tapped, obtaining from the upper one a light fluid, and from the lower one a much darker fluid. On account of her age no vaginal examination was made. The fluids pointed strongly toward an ovarian cystoma. I again advised an exploratory incision.

April 29, 1893. The patient was finally brought to the Jefferson College Hospital. She has been tapped twice since February, 1892, the last time in February, 1893, when six and a half gallons were drawn off. She is now enormously swollen. The measurements are as follows: From the ensiform to the umbilicus, sixteen and one-half inches; from the ensiform to the pubes, twenty-nine and one-half inches (this measurement in myself reaches from the ensiform to the middle of the calf of my leg); circumference, forty-nine inches. The veins over the abdomen are very large. Nothing can be made out in the interior in consequence of the enormous abdominal distension. Examination of the urine shows no albumin, and a very slight trace of sugar (?).

*Operation.* April 30, 1893, a small incision was made in the median line above the umbilicus, as the greater mass of the tumor lay there. A large trocar was thrust in and evacuated a very large quantity of characteristic opalescent ovarian fluid. The escape of this fluid revealed through the abdominal wall large masses lying especially under the liver and in the right iliac fossa. After this evacuation I enlarged the incision until it measured eventually about eight inches in length. I introduced my hand and found an enormous ovarian cyst, reaching up to the diaphragm, and pushing everything out of its way. There were a number of moderate adhesions, chiefly to the belly wall and the omentum. The viscera were fortunately entirely free. The pedicle was only two and one-half inches broad. The tumor arose in the right ovary, the left ovary being healthy but small.

The weight of the solid mass removed was twenty-seven pounds, and by actual weighing the fluid removed weighed eighty-four pounds, making a total of 111 pounds. The child herself weighed but sixty eight pounds.

After the removal of the tumor I never saw so curious a looking abdominal cavity. It looked almost like that of an eviscerated cadaver in the dissecting-room. The tumor had so pushed the liver to the right and backward, and the stomach to the left, that nearly the whole of the diaphragm was exposed, and flapped up and down with the pulsations of the heart. Down the middle of the cavity the bodies of the vertebra were entirely exposed, showing the aorta and vena cava to their bifurcations, the intestines being a very minor consideration and pushed to each side in the hollow of the ribs and the lumbar region. When the abdominal wall was sutured the abdomen was excessively scaphoid, the anterior abdominal wall lying directly on the aorta and vertebræ. The puckering of the skin, although moderately marked, was much less than I had expected.

When the operation was completed a glass drainage tube was inserted, and she was put to bed in very fair condition, in view of the gravity of the operation. The tumor was a multilocular cyst.

May 18, 1893. The child has made an uninterrupted recovery. The drainage tube was removed on the fifth day, when the discharge had become almost nothing, but three days later a slight rise of temperature took place, and the discharge recommenced. A small rubber drainage tube was therefore reinserted for a few days. She sat up at the end of two weeks, and will go home as soon as the slight discharge from the drainage opening ceases.

*Remarks.* I have not had time to search through the literature of ovariotomy, but so far as my memory serves I have never known a larger tumor removed from a child. It weighed just one and a half times as much as the patient. Her recovery has been most satisfactory in spite of a very poor and capricious appetite. The chief lesson the case teaches is the value of an exploratory incision in every case of doubt. Had this been done, instead of a mere tapping, in October, 1891, when the tumor was much smaller, the prognosis would have been much more favorable, and she would have been spared a year and a half of needless suffering. What seemed to be a most formidable operation really proved to be almost a simple one, the adhesions and the pedicle being most favorable for the speedy recovery which has ensued.

